# Emergence of Nanotechnology to Fight HIV Sexual Transmission: The Trip of G2-S16 Polyanionic Carbosilane Dendrimer to Possible Pre-Clinical Trials

**DOI:** 10.3390/ijms21249403

**Published:** 2020-12-10

**Authors:** Ignacio Relaño-Rodríguez, Maria Ángeles Muñoz-Fernández

**Affiliations:** 1 Head Section of Immunology, Molecular Immunology Laboratory, General Universitary Hospital Gregorio Marañón, C/Dr. Esquerdo 46, 28007 Madrid, Spain; ignaxete@hotmail.com; 2 Health Research Institute Gregorio Marañon (IiSGM), C/Dr. Esquerdo 46, 28007 Madrid, Spain; 3 Spanish HIV HGM BioBank, C/Dr. Esquerdo 46, 28007 Madrid, Spain; 4 Networking Research Center on Bioengineering, Biomaterials and Nanomedicine (CIBER-BBN), C/Dr. Esquerdo 46, 28007 Madrid, Spain

**Keywords:** nanotechnology, G2-S16 PCD, HIV prophylaxis, sexually transmitted infection, microbicide

## Abstract

Development of new, safe, and effective microbicides to prevent human immunodeficiency virus HIV sexual transmission is needed. Unfortunately, most microbicides proved ineffective to prevent the risk of HIV-infection in clinical trials. We are working with G2-S16 polyanionic carbosilane dendrimer (PCD) as a new possible vaginal topical microbicide, based on its short reaction times, wide availability, high reproducibility, and quantitative yields of reaction. G2-S16 PCD exerts anti-HIV activity at an early stage of viral replication, by blocking gp120/CD4/CCR5 interaction, and providing a barrier against infection for long periods of time. G2-S16 PCD was stable at different pH values, as well as in the presence of seminal fluids. It maintained the anti-HIV activity against R5/X4 HIV over time, did not generate any type of drug resistance, and retained the anti-HIV effect when exposed to semen-enhanced viral infection. Importantly, G2-S16 PCD did not modify vaginal microbiota neither in vitro or in vivo. Histopathological examination did not show vaginal irritation, inflammation, lesions, or damage in the vaginal mucosa, after administration of G2-S16 PCD at different concentrations and times in female mice and rabbit animal models. Based on these promising data, G2-S16 PCD could become a good, safe, and readily available candidate to use as a topical vaginal microbicide against HIV.

## 1. Introduction

Joint United Nations Program on HIV/AIDS (UNAIDS) estimates that 37.9 million people were living with HIV worldwide at end of 2018 and the fastest growing phase of this pandemic is currently related to heterosexual transmission in women. HIV sexual transmission infection (STI) is held accountable for around 80% of all infections, with roughly half of the affected individuals being women [1,2]. Sexual transmission is responsible for the majority of HIV infections due to sexual contact with infected cervicovaginal secretions or semen containing HIV-infected CD4 T-lymphocytes [3,4,5,6]. Although female genital mucosa is a major portal for entry of HIV into the body, accounting for initiation of 40% global HIV infections, acute events that follow HIV exposure in female genital tract (FGT) still remain unclear [7]. Moreover, the rate of HIV transmission is much greater from males to females than it is the other way around. This difference in HIV transmission, along with other biological factors, likely explains higher incidence of STI found in women [8]. The actual basis for controlling HIV/AIDS epidemic depends on the development of new prevention methods and strategies, due to the failures in synthesizing an efficient vaccine [9,10]. Self-administered topical microbicides could be useful for all risk groups to decrease HIV infection rates [11,12].

Previous publications showed that microbicides are classified as semi-solid (creams, gels) and solid (films, suppositories, intravaginal rings) formulations that could be applied to vaginal or rectal mucosa to attack viral or cell targets, and prevent, or at least reduce, HIV transmission and infection [13]. Briefly, HIV-infected genital fluids, such as cervicovaginal secretions or semen, could release HIV virions in semen and HIV-infected cells [14,15]. Human vaginal tissue ex-vivo experiments showed HIV virions uptake across vaginal epithelium, starting from primary HIV infection. Once in contact with vaginal mucosa, HIV penetrates the epithelium to reach CD4 T-cells, macrophages (MØ), or dendritic cells (DC), located in the submucosa [16,17]. Male cells or male soluble factors can activate the female epithelial surface. At this point, defenses against HIV include physical barriers (cervical mucus), antiviral factors secreted by innate immune system, and protective factors such as vaginal microflora dominated by *Lactobacilli* spp. that preserve an acidic environment in vaginal vault [18]. HIV potentially evades the host defenses by making use of impaired epithelial integrity states caused by microtrauma or physical abrasion, ulceration, inflammation, certain hormonal status or low micronutrient levels, resulting in access to submucosa [19,20]. Therefore, HIV infects local immune cells such as T-cells, MØ, or DC, followed by early dissemination from genital tract-associated mucosa to regional lymph nodes [21,22].

As previously described in the available bibliography, microbicide prevention is rooted in the use of drugs or compounds that directly inactivate HIV in vaginal fluids or in semen, or alternatively, impede HIV integration target cells found in vagina or rectum [23]. An effective microbicide must prevent HIV infection in several target cells (CD4-expressing, CD4-non-expressing), as well as in the presence of concurrent vaginal infections due to other viral, bacterial, parasitic, or fungal microorganisms [24,25,26].

**Figure 1:**
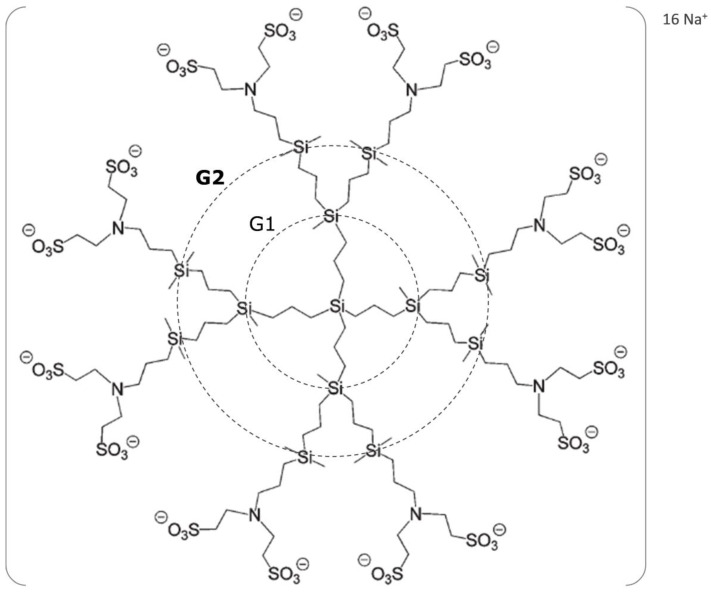
Chemical structure of second-generation polyanionic carbosilane dendrimer G2-S16. The generation were defined as the number of repeated layers of silicon atoms forming the dendrimer. The capping layer consists of 16 sulfonate groups (−SO_3_^−^). The molecular formula is C_112_H_244_N_8_Na_16_O_48_S_16_Si_13_ and the molecular weight is 3717.15 g/mol.

This review analyzes the advances in the state of the art of G2-S16 PCD (Figure 1) as a possible innovative and promising microbicide close to reaching clinical trials [27,28]. G2-S16 PCD provides remarkable information on the future growth of multi-purpose interventions to impede HIV infection in women.

## 2. Antiviral Microbicides

Progress of microbicides is based on the use of antiretrovirals (ARV) that precisely act against the HIV lifecycle. Dapivirine (DPV), which binds allosterically to HIV reverse transcriptase, prevents HIV replication and is one of the leading drug candidates in the field. It is currently being tested in various dosage forms and several possible presentations such as gel, film, or intravaginal ring were analyzed [29,30,31,32,33,34,35]. DPV vaginal ring can be employed in combination with a wide range of hormonal contraceptive methods, without compromising the contraception effectiveness [31]. Women enrolled in MTN-020/ASPIRE, a randomized, double-blind, placebo-controlled phase III clinical trial (Clinicaltrials.gov NCT01617096) for checking the safety and effectiveness of DPV vaginal ring for HIV prevention, manifested the appearance of side effects. These results included inflammation, reddening, or swelling of the cervix, urinary tract infection, loss of bladder control, headache, pain during sex, and pelvic pain [31,32,36,37]. Furthermore, the risk of developing resistance to this ARV is high, especially in cases of intermittent or noncompliant use. Sexual transmission of ARV-resistant HIV is a known fact. However, its clinical significance remains questionable. It is unknown whether high levels of DPV in genital tract achieved with intravaginal DPV ring are sufficient to prevent infection by Non-nucleoside reverse transcriptase inhibitors (NNRTI) resistant variants. DPV vaginal ring is still being studied, and the whole repertoire of feasible good or side effects is not completely analyzed.

Reverse transcriptase inhibitor, tenofovir (TFV), is one of the most studied microbicides for topical vaginal application against HIV and HSV-2. During bacterial vaginosis, the rapid depletion of TFV by various anaerobic bacteria, such as *Gardenella vaginalis,* supplies a biological mechanism included in a multifactorial process, causing increased vaginal inflammation and adherence [29,34,38]. The first successful phase II clinical trial of an ARV-based microbicide (CAPRISA 004, Centre for the AIDS Program of Research in South Africa) used a TFV-based gel to prevent man-to-woman HIV transmission. This clinical trial showed that ARV could be effective when used as a topical microbicide, reducing HIV infection by 39% in South African women. However, high adherence was likely required for ARV to be effective, and vaginal microbiota modulated its efficacy. More recent failures of TFV in other microbicide clinical trials, such as the VOICE study (Vaginal and Oral Interventions to Control the Epidemic) [39,40], propose that there is still more room for improvement and efforts are needed to develop new effective microbicide strategies [39].

One of the main barriers for microbicide success, as shown by Zirafi et al. is based on the fact that semen decreases sensitivity of HIV to the nucleotide, NNRTI, or integrase inhibitors acting on intracellular targets [41]. This is caused by prostatic acid phosphatase (PAP), a protein of the semen secreted by the prostate gland [42]. The effect of PAP revolves around amyloid fibrils, which causes so-called semen-enhanced viral infection (SEVI). These SEVI capture HIV virions, thus, promoting their adhesion to target cells and increasing their ability to infect human cells, regardless of the cell type involved [43,44,45]. Reports specify that exposure of HIV particles to a concentration of 10% semen increases HIV infectivity up to 10-fold, and also shortens the exposure time to microbicides acting outside of the cell, which aim to avoid virus entry by impeding accessibility to virus membrane and glycoproteins [41,45,46,47,48]. This might explain why many microbicides that show high efficacy in vitro fail to be effective in clinical trials, and thereby, no commercially topical microbicide is available yet [49].

In summary, the main weaknesses of microbicides are—variability of their features, adherence needs, emergence of virus resistance against them, need for a high concentration so that they are effective, side effects derived from their toxicity (such as vaginal inflammation or irritation, microbiota alteration, among others), failure to prevent semen-exposed HIV infection, high daily cost, need to use them as a lifelong medication, and possible drug–drug interactions that might arise.

## 3. Why Is a New Nanomicrobicide Candidate Needed?

Nanotechnology provides new and suitable approaches to acquire novel, potent, and safer antiviral agents, such as PCD [50,51,52,53]. PCDs are a group of well-defined hyper-branched polymers with a nanoscale globular shape, well-defined functional groups located at the periphery and internal cavities that are able to encapsulate guest molecules. The reasonable manufacture cost and controlled synthesis of PCD, along with their high biocompatibility, solubility, reactivity, low polydispersity, and polyvalency, differentiate them from standard linear polymers [54]. PCDs are especially suitable for therapeutic approaches, due to their simple synthesis, allowing us to obtain large amounts of polymer, with a defined molecular weight and a specific number of functional terminals to be generated. Moreover, their biochemical stability, biological inertness, and low polarity of the C–Si bond confers dendrimers many capabilities for their use as HIV microbicides [55,56,57,58].

PCDs with functionalized groups at their periphery can bind to their target, providing a polyvalent strategy for the development of strong viral entry inhibitors. The only topical nano-microbicide that entered a phase I clinical trial for intravaginal microbicides against HIV is poly(L-lysine) (PLL)-dendrimer SPL7013, the active compound of VivaGel™ [59]. However, lack of broad anti-HIV activity against R5-HIV isolates [60], lack of adherence, evidences of inflammation, cytotoxic effect after repeated vaginal use, ineffectiveness due to the fact that SPL7013 was 18- to 21-fold less active against semen-treated virus and emergence of resistances are the main limitations of VivaGel™ [41,61,62,63,64,65].

More sophisticated PCDs acting as HIV entry inhibitors that could prevent non-specific HIV binding to host cells are under development as vaginal microbicides. These PCDs act by blocking different molecules, such as heparan sulfate proteoglycans (HSPG), glycosphingolipids, or CD4/gp120 interactions, DC-specific intercellular adhesion molecule-3-grabbing non-integrin (DC-SIGN), among other mechanisms [51]. Further investigations must be performed for the development of a functional microbicide, as ideally, these PCD should not only prevent HIV infection but also other STI.

## 4. Why Is G2-S16 Dendrimer Emerging as a New Potential Vaginal Topical Microbicide?

Our group performed an impartial, cell-based screening assay for 74 antiviral nanocompounds to determine their potency and cytotoxicity against HIV, HSV-2, and other STI. These nanocompounds included 28 stable PCDs, 27 polyanionic carbosilane dendrons, 11 “bowtie”, 6 gold nanoparticles functionalized with polyanionic carbosilane dendrons, and 2 iron oxide nanoparticles. Primary screening at 1–100 µM concentration determined that 21 of these compounds were highly active and non-toxic, while two compounds were ruled out for further studies, due to their significant cytotoxicity. Given the number of primary hits, we set out the counter screen for these 19 candidates to prioritize them by calculating their potency (EC_50_) and toxicity (CC_50_) indices. From this initial selection, we selected G1-S4, G2-S16, G2-S24P, and G3-S16 dendrimers, as they showed the lowest EC_50_ values and highest selectivity indices. The selected dendrimers displayed noteworthy antiviral activity (EC_50_ < 300 nM) with practically no cytotoxicity associated, as toxicity started appearing at significantly higher concentrations. Although, we are currently working with 4 selected PCD, G2-S16 dendrimer showed the most potent EC_50_ among all PCDs. The potent anti-HIV activity of G2-S16 PCD (C112H244N8Na16-O48S16Si13; molecular weight, Mw: 3717.2 g mol^−1^) was previously reported [56]. Concretely, G2-S16 PCD with a carbosilane structure containing 16 sulfonate peripheral groups derived from a Si(C3H5)4 silicon core was selected [55]. G2-S16 PCD was selected as a new possible vaginal topical microbicide, due to its short reaction times, easy availability, high reproducibility, and quantitative yields of reaction. Its peripheral anionic groups make this PCD water soluble, despite its highly hydrophobic framework. G2-S16 PCD presents a net negative charge in water, influenced by the medium pH (Table 1).

Due to the fact that cervical and foreskin epithelia are the places affected by STI, G2-S16 PCD toxicology studies in these areas are mandatory. For achieving that objective, animal models and human clinical trials require large amounts of compound to validate its efficacy. It is important to know that although the scale-up of G2-S16 PCD is being analyzed by a biotechnological company, the manufacture and scale-up requirements of nanoparticles are key factors that need to be thoroughly studied in the early development steps of microbicides. This step is crucial, as a very active compound might be developed, but chances to reach clinical trials is negligible if its synthesis is not scalable. Moreover, all nanoparticles should be labelled with different probes for biodistribution studies. G2-S16 PCD was already labelled with a fluorophore [56].

## 5. How Does G2-S16 PCD Work In Vitro?

### 5.1. Biocompatibility

Since the microbicide compound will be in direct contact with vaginal epithelium, the safety and biocompatibility of compounds must be checked. In the case of G2-S16 PCD, this assessment was performed in human epithelial cell lines derived from uterus (HEC-1A and HeLa), human vagina (VK2/E6E7), ectocervix (Ect1/E6/E7) and endocervix cells (End1/E6E7), and in primary human cells (such as PBMC, CD4 T lymphocytes, Treg, monocytes, MØ and DC) [57,66,67]. It proved to be safe at different times with increasing concentrations of G2-S16 PCD, using the MTT assay (3-(4,5-dimethyl-2-thiazolyl)-2,5-diphenyl-2H-tetrazoliumbromide) and the LDH assay for quantifying cytotoxicity, based on measurement of the activity of lactate dehydrogenase or 7AAD FACS assay in vitro. Compounds are considered to be toxic when the survival rate of cells is higher than 80%. G2-S16 PCD was considered non-toxic at 20 μM in CD4 T lymphocytes, monocytes, DC, and MØ; at 50 μM in PBMC, Ect1/E6/E7 and End1/E6E7 cells; and at 100 μM in TZM-bl, HeLa, and VK2/E6E7 cell lines. G2-S16 PCD showed a great biocompatibility in all type of cells studied (Table 1).

### 5.2. Immune System

Genital epithelial cells (GEC) are the first cells in FGT that HIV encounters during sexual transmission. GEC are specialized in recognizing and responding to incoming pathogens, immediately initiating the innate immune responses that constitute the second line of defense in the FGT [68,69,70]. The early interactions between HIV and mucosal epithelium showed that GEC recognize and respond to HIV/gp120, by causing and secreting pro-inflammatory cytokines, including TNF α and IL-6, and by allowing microbial translocation. Thereby, strategies to prevent barrier loss following exposure to HIV/gp120, provide the basis for prophylactic treatments trying to prevent immune activation during HIV infection.

Previous studies propose that dissimilarities in the capacity to answer to microbial products can reach various levels of TLR-mediated activation. TLR trans-membrane proteins are a highly conserved family of pathogen-associated molecular pattern (PAMP) receptors, specialized in detecting foreign material [71,72]. These receptors enable TLR-expressing cells to respond to xenocompounds connected with non-self PAMP, and accordingly recognize and respond to invading pathogens. TLRs also play an important role in the beginning of innate immune response to infections, and following adaptive immune response also help shape the third line of defense [73,74]. We selected TLR2 and TLR4, as both are primarily responsible for glycoproteins recognition at cell surface and for initiating intracellular cascade that results in HIV activation. Normal epithelial components should not cause inflammation. Several assays were performed to study the modulation of TLR2 and TLR4 expression in VK2/E6E7 cell line, iDC, MØ, and CD4 T-lymphocytes exposed to 3 h (early time) and 18 h (late time), with G2-S16 PCD treatment. Although G2-S16 PCD inhibits >90% HIV infection, it did not stimulate the expression of TLR2 and TLR4 at 3 h and 18 h, and consequently, did not activate inflammatory cytokines. Nonetheless, MØ with proinflammatory phenotype played a key role in the development of immune response against pathogens via antigen presentation, secretion of microbicidal products, and production of inflammatory mediators. We showed that G2-S16 PCD did not affect TNF-α and IL-6 cytokines production in MØ. Nonetheless, as expected, G2-S16 PCD decreased the expression of TNF-α in presence of LPS. G2-S16 PCD did not modify the functional capacity of MØ, thus, enhancing its value as a microbicide, due to the fact that, in the presence of pathogens (for example, HIV), G2-S16 PCD decreases the expression of TNF-α in vaginal mucosa and reduces or blocks infection. Thereby, G2-S16 PCD did not affect the production of CCL3 and CCL4 chemokines in MØ, suggesting that treatment with G2-S16 PCD might not induce infiltration of cells in inflamed tissue. G2-S16 PCD can be used as a biologically safe agent without promoting inflammation, and can even slightly decrease the production of some cytokines. G2-S16 PCD did not modify the expression of CD4, CD8, CCR5, and CXCR4 receptors, which was relevant, as HIV especially infects CD4 T cells and activated CD4 T cells expressing α4 β7 or α4 β1 in the presence of CCR5 or CXCR4 coreceptors. Different concentrations of G2-S16 PCD did not induce activation or increased proliferation of PBMC [50,57].

The cytokine profiles VK2/E6E7 epithelial cell line and PBMC treated with G2-S16 PCD at early and late times, were assessed with a Th1/Th2 inflammation assay (IL-12, TNF-α, IL-17A, IL-4, IL-6, IL-8, IL-10, IL-2, and IFN γ). G2-S16 PCD did not activate this cellular inflammatory pathway and did not induce changes in the expression levels of a wide array of inflammatory-related genes, which is another factor to take into account when designing and developing a safe topical microbicide [17,53]. These data clearly revealed that this G2-S16 PCD did not induce an inflammatory microenvironment. Our data clearly showed that the treatment of epithelial cells and PBMC with G2-S16 PCD, did not make changes in activation of PBMC or in proinflammatory cytokines profile (Table 1). Moreover, we performed several assays to investigate the impact of G2-S16 PCD in the vagina immune system. Our results demonstrated the biosafety of this G2-S16 PCD microbicide, as it did not modify the normal activity of the main immune system cells [75].

### 5.3. Vaginal Enviroment

pH of healthy FGT is acid (pH: 4.0–5.8), while pH of normal semen is alkaline (pH: 8–8.5). Thus, ensuring that G2-S16 PCD could be effective in an alkalinized environment after sexual intercourse is crucial. G2-S16 PCD is stable in a wide pH range of 3–8.5 and maintains a stable anti-HIV activity against different R5 and X4 HIV strains, overtime in seminal fluids [17]. Sperm cultured in presence of G2-S16 PCD (10 and 50 μM) after 100s post-treatment, retained 70% of sperm motility on samples of human spermatozoa. Sperm motility and various bacteria present in normal vaginal flora showed very similar response patterns, compared to those found in the untreated control, indicating that G2-S16 PCD is not a spermicidal nor anti-bacterial compound [76]. In this context, G2-S16 PCD in combination with Platicodin D, a spermicide compound, could prevent HIV infection and non-desired pregnancies at the same time. Our results showed that a combination of G2-S16 PCD/Platicodin D inhibit 95% HIV infection and retain 100% of sperm mobility, not showing toxicity, neither in vitro nor in vivo [77].

Vaginal microflora regulates epithelial innate immunity in a species- and strain-specific manner, and topically applied microbicides should not alter the delicate homeostatic balance between epithelial components and microbiota. Normal vaginal microbiota contains a wide variety of species that maintain an acidic pH, by producing hydrogen peroxide and lactic acid [78]. Changes in this ecosystem cause several vaginal infections, overgrowth of anaerobes, and a noticeably increased risk for acquisition of HIV infection [79]. G2-S16 PCD impact against an array of 29 bacterial species, typically found in gut and vaginal flora, as well as some pathogenic bacteria, were assessed to determine the minimal inhibitory concentration. We did not observe a negative effect on any bacterial species studied, including *Lactobacillus acidofillus,* which is an abundant beneficial species in the intestinal tract and vagina. Lactobacilli contribute to immunity in FGT by providing a non-specific protection against a broad, large range of pathogens [79]. Bacterial cultures grew normally, even when exposed to 250 μM of G2-S16 PCD. Bacteria present in normal vaginal flora showed indentical response patterns, compared to those in the untreated control, clearly showing that G2-S16 PCD is not an antibacterial compound [53]. G2-S16 PCD is a potential candidate to create a frontline biological barrier against viruses, as this dendrimer does not show any negative effect on the innate immune system and normal vaginal flora (Table 1).

### 5.4. Antiviral Activity

G2-S16 PCD has multifactorial and non-specific function and acts as a virucidal agent inhibiting viral entry, providing a barrier against infection for a long time and blocking the spread of HIV from cell-to-cell (CTC) [53,55,56,66]. Given that semen is the major vector for general spread of HIV infections, inhibition experiments were performed with G2-S16 PCD, up to a maximum non-toxic concentration of 20 µM, in the presence of amyloid fibrils in semen against T/F pCH058 and pTHRO R5-HIV viruses and the R5-HIV_NL(AD8)_ laboratory strain. G2-S16 PCD clearly showed inhibitory results on HIV infection at non-toxic concentrations in TZM.bl cells and PBMC. This dendrimer reduced the rates of HIV infection, not only by neutralizing the negative charges of the virus, but also by creating a competitive binding to viral targets (gp120 complex) and to cellular targets (CD4 receptor in areas relevant to CD4/gp120 and CCR5/gp120 or CXCR4/gp120 interactions). Antiviral activity of its sulfonate end-groups was not modified by semen components, by the alkaline pH of semen [80,81,82,83,84], or by competitive binding to viral env-glycoproteins [85].

The env-glycoproteins of HIV are the crucial elements involved in HIV entry. The establishment of HIV infection in a naïve host most often results from transmission and successive propagation of a single virus strain, termed as T/F virus [86]. T/F viruses mostly show CCR5-(R5) tropism, infect CD4 T-cells and share certain genetic features, including shorter variable loops, fewer potential N-linked glycosylation sites and amino acid signatures, which might affect env-glycoprotein surface expression or other viral properties [87,88,89,90]. HIV inhibitory effect of G2-S16 PCD was >90% in PBMC. This effect was not only observed in pTHRO.c and pCH058.c R5-HIV T/F viruses [91], but also in the most prevalent R5-tropic HIV clinical subtypes, such as R5-HIV_R23_ (clade A1), R5-HIV_X-1936_ (clade C), R5-HIVX_2160-2_ (clade G), R5-HIV_ESX-3016-2_ (clade F1), R5-HIV_ESP-2392-3_ (clade CRF02_AG), and R5-HIV_ESX-2457-2_ (clade CRF47_BF) isolates [53], as well as in R5 HIV_NL(AD8)_, R5 HIV_WT/BAL_, R5-HIV-2_HSM2.03_, R5-HIV-2_HCC12.3,_ X4 HIV_NL4.3_, X4-HIV-2_XT03_, X4-HIV-2_X4-CC10.3_, and dual tropic HIV_89.6_ laboratory strains [91]. Summarizing, G2-S16 PCD shows a high anti-HIV activity in urogenital epithelial cells against T/F HIV strains, R5-tropic clinical subtypes, and X4 laboratory strains. G2-S16 PCD reduces HIV infection when the pre-treatment of cells is performed at an early time and the inhibitory effect is prolonged overtime (Table 1).

The capacity of mucosal epithelial cells to maintain an intact, polarized monolayer when treated with a microbicide, is one of the crucial steps to differentiate safe nanoparticles. Cell-free and cell-associated viruses are prevalent infectious forms of HIV present in semen and cervico-vaginal secretions [92,93]. G2-S16 PCD prevented the transmission of cell-free and cell-associated HIV, through the epithelial barrier [53,55]. G2-S16 PCD does not just block activated PBMC infection with HIV strains, quickly after its application, but also partially inhibits HIV transmission through the epithelial monolayer, and blocks subsequent HIV infection of PBMC. G2-S16 PCD is able to protect the integrity of monolayer, and protects urogenital epithelial cells from the tight junction disruption induced by HIV (Table 1).

As aforementioned, adult cervical and foreskin epithelia serve as an entry site for sexual transmission of HIV [94,95,96]. The three-dimensional full thickness EpiVaginal™ (VEC-100) (Mattek Corporation; Ashland, MA, USA) ectocervical tissue model is a highly differentiated structure, which parallels in vivo tissue and is ideal for toxicity studies on feminine hygiene, vaginal care, and microbicide products. EpiVaginal™ is gradually replacing the traditional in vivo models to study vaginal irritation, due to its high reliability and reproducibility. Our G2-S16 PCD was nontoxic at concentration of 500 μM and this EpiVaginal™ (VEC-100) ectocervical tissue model showed the safety of G2-S16 PCD for vaginal application to control viral transmission [67] (Table 1).

Development of resistance to nanocompounds and ARV entry inhibitors is one of the main problems when testing microbicides in human clinical trials. Treatment with G2-S16 PCD against HIV infection for thirty successive cells passages in MT-2 cells, did not generate any type of drug resistance. Even more, efficacy and thigh genetic barrier of G2-S16 PCD was also demonstrated, thus, reinforcing the evidences for its security and effectiveness [97] (Table 1).

## 6. Which Is the Mechanism of Action of G2-S16 PCD?

One of the most promising targets within HIV lifecycle is the viral entry/fusion process [56], which is divided into three steps—(i) attachment of gp120 to CD4, (ii) binding to CCR5 or CXCR4, and (iii) fusion of env-glycoprotein with cell membrane, followed by the release of viral capsid into the cytoplasm of the host cell [51]. Viral entry is a process that involves the binding of a virus to cell surface, its fusion to cell membrane, and integration of viral genome into target cells. Polyamidoamine (PAMAM) dendrimers weaken the CD4/gp120 complex and alter the dissociation pathway of the complex, thus, inhibiting the HIV entry into target cells, as well as modulating the interactions of hydrophobic and hydrophilic residues between gp120 with CD4, altering the hydration of hydrophobic interfacial cavity, and disrupting hydrogen bonds across gp120 and CD4, leading to a modification of their patterns of dissociation [98]. However, our results clearly indicated that G2-S16 PCD inhibits HIV infection by blocking viral entry, particularly acting at the gp120/CD4 interaction. The cell-based fusion assay with G2-S16 PCD showed a better inhibitory effect on host CD4 cells than on env-expressing cells. These data, combined with a gp120-capture ELISA, support the hypothesis that G2-S16 PCD inhibits HIV infection, by blocking the HIV entry, mainly by hindering the gp120/CD4 interaction. G2-S16 PCD inhibition mechanism against HIV could likely be related to a direct viral inactivation, without excluding the possible ability of G2-S16 to block CD4 receptors at surface of target cells. The V3 loop of gp120 stands out as the main candidate for G2-S16 PCD binding, due to the ability of the anionic charges of this PCD to interact with gp120-positive charges. This binding is higher when HIV strains are used than when HIV-2 strains are involved, due to variation in amino acids present in the V3 loop of gp120 [55,99]. In addition, Chonco et al. and Sepulveda-Crespo et al. corroborated that G2-S16 PCD forms stable complexes with gp120, CD4 receptors, and to a lesser extent, with CCR5 and CXCR4 co-receptors [99,100]. We studied different combinations with actual antiviral compounds, G2-S16 dendrimer showed good compatibility with either maraviroc, tenofovir, or a combination of both [58,101,102]. Molecular modeling dynamics simulations of G2-S16 PCD interaction with gp120 and CD4 confirmed that, although G2-S16 PCD binds to CD4 receptor at important areas for gp120/CD4 and gp120/CCR5 or CXCR4 docking, computational results indicated a significantly higher binding affinity in the case of the G2-S16 PCD/gp120 complex [103].

In essence, G2-S16 PCD interacts non-specifically with gp120 and the cell receptors, in a dose-dependent manner, although it does so predominantly with the CD4 receptors of the host cells. Previous studies using molecular dynamics simulations, support this idea, because the mechanism of action of dendrimer is related to electrostatic interactions between the anionic functional groups located at dendrimer periphery and glycoproteins of viral envelope or proteins at host cell surface that are involved in HIV infection. Our data clearly suggest that G2-S16 PCD could protect urogenital epithelial cells from the tight junction disruption induced by HIV infection.

SPL7013 dendrimer, the active product of VivaGel™, is not a virucidal agent, as it lacks a broad antiviral activity against R5-HIV isolates, due to the fact that V3 loops vary in positive charges between the R5- and X4-HIV isolates. We obtained several lines of evidence that other effective mechanisms of inactivation can be involved. G2-S16 PCD not only binds to the V3 loop and co-receptor binding sites [55], but can also interact with two disulfide bonds located on the gp120, thus, modifying this viral protein by denaturing its disulfide-bonded domain. Due to the antiviral effect of G2-S16 PCD being increased with incubation time, G2-S16 PCD might initially bind to the gp120 knobs, and then inhibit HIV infection by irreversibly modifying these viral structures. HIV particles tend to be susceptible to the G2-S16 PCD virucidal activity through a mechanism that does not involve disruption of the integrity of HIV capsid, destabilizing the core-membrane linkage or removing gp120 from the viral surface, through shedding. Complete inactivation makes us think that inhibition of HIV particles by the G2-S16 PCD is caused by irreversible or tight binding to HIV env-glycoproteins, physically blocking the binding of virus to target cell receptors, or by creating strong interactions that destroy the HIV infectivity. Another possibility is that virus-cell fusion occurs with the endosomal membrane, following endocytic uptake of virus particles [104]. All alternatives of HIV entry probably coexists, due to the fact that G2-S16 PCD inhibits binding but does not completely inhibit virus internalization. The HIV capsid protein can be altered by G2-S16 PCD during the passage to cell and this leads to problems in its disaggregation. Most likely, p24 Ag enters the cell but retrotranscriptase, integrase, and viral RNA do not. Consequently, the HIV particles are not infectious. Ability of G2-S16 PCD to directly inactivate R5-HIV_NLAD8_ and X4-HIV_NL4.3_ isolates and its HIV virucidal activity were evaluated [100].

A safe microbicide should remain effective for many hours after gel application, even after the gel is washed away. First-generation microbicides are only effective for a few hours, and consequently, require administration shortly before sexual intercourse [105]. In contrast, we have showed that treatment with G2-S16 PCD for 1 h, reduced the transmission of HIV infection by 80% in a tropism-independent manner, an effect that lasted for 24–48 h. In agreement with other microbicide candidates [106], the exposure of uninfected cells to G2-S16 PCD conferred a protection for long periods of time and provided a refractory effect to a subsequent HIV infection, thus, minimizing the HIV sexual transmission from infected to non-infected individuals. Our results indicate that G2-S16 PCD irreversibly targets HIV virions, but without completely destabilizing the viral capsid.

In order to show the antiviral target of G2-S16 PCD, a time-of-addition assay was performed to delimit the stage(s) of HIV lifecycle that get blocked by G2-S16 PCD, compared to inhibitor T-20 (that decline after 2 h), AZT (that decline after 5 h), and raltegravir (RAL) (that decline after 7 h post-infection). G2-S16 PCD retained its antiviral activity for up to 2–3 h after HIV infection, showing a way of action similar to that of T-20. These findings, consistent with those obtained for other polyanionic compounds [107], confirmed that G2-S16 PCD inhibits HIV infection at the initial stages of HIV lifecycle, probably by inactivating the HIV particles or interfering with the recognition of receptors at the surface of target cells, a process named viral entry/fusion [55].

G2-S16 PCD prevents HIV entry in a manner that is independent of the number of viral particles or the presence of semen, and it also acts as an inhibitor of CTC transmission [55,57]. CTC transmission is the most effective pathway of spreading HIV and G2-S16 PCD is able to block the CTC HIV spread. The proposed mechanisms of Raji-mediated HIV transmission suggest three possible transmission ways: (1) HIV infects the target cells and replicates and produces virions that are released to infect new target cells (cis-infection) [108]. (2) Trans-infection, where HIV is retained at the cell surface of donor cells and transmitted to the target cells through contact, and formation of a virological synapse [109]. (3) Transmission by exosome secretion [110]. CTC transmission is 100–1000-fold greater than a cell-free viral infection [111]. HIV-pulsed DC efficiently transmits HIV to the co-cultured CD4 T cells that were used to study the mechanism underlying CTC transmission [112,113]. CTC protects viruses from humoral immune response and antiviral treatments, enables a residual replication, and establishment and maintenance of viral reservoirs. G2-S16 PCD efficiently prevents HIV CTC infection, albeit at a higher concentration compared to that effective against cell-free infection. However, infected cells expose virus-encoded fusion proteins on their surface as a consequence of the HIV replicative cycle. These fusion proteins create interactions with the noninfected cells through the CD4 receptor and the CXCR4 co-receptor, leading to formation of giant multinucleated cells known as syncytia. G2-S16 PCD, not only significantly inhibits the X4-HIV infection on MT-2 cells, but also significantly reduces syncytia formation in an HIV env-mediated CTC fusion model.

## 7. How Does G2-S16 Dendrimer Work In Vivo?

### 7.1. Biocompatibility

Topical effect of G2-S16 PCD on mucosa was first studied in CD1 (ICR) mice for in vivo evaluation, analyzing the integrity of mucosal tissue [53]. No mortality or sign of vaginal discharge, erythema, edema, irritation, or inflammation at the lowest dose (5 mM) or a higher dose (50 mM) of G2-S16 PCD were detected. No characteristic abnormalities were found during the histopathological analysis of vaginal tissues, compared to the negative control [53,67,99,114]. Moreover, G2-S16 PCD was intravaginally applied for 7 consecutive days in BALB/c mice, at different doses and concentrations, based on the available literature [115], and pathological examinations of vaginal tissues were performed. In post-mortem and histopathological examinations, G2-S16 PCD did not cause disruption of epithelial cells and did not produce damage in vaginal mucosa [51,116]. Moreover, due to the grade of histopathological similarity between rabbit and human vaginas, female rabbit was also selected as an animal model. No mortality or signs of vaginal discharge, erythema or edema, irritation, inflammation or vaginal lesions in New Zealand white female rabbits studied were detected (Table 1) [55]. Semen, cervicovaginal secretions, and normal flora must be included in studies of G2-S16 PCD activity to mimic real live conditions occurring in HIV sexual transmission. In this context, G2-S16 PCD did not trigger inflammation or vaginal irritation in mice and rabbits. These features are important and need to be considered, since creating an inflammatory state might fatally increase viral transmission. Although rodents are the standard animal models for evaluating toxic drugs, the zebrafish embryo is emerging as an important tool for toxicity testing and for carrying out fast reproducible tests [117,118]. No significant differences were found in mortality, sub-lethal, or teratogenic effects, when the embryos were treated with G2-S16 PCD, as compared to the controls.

### 7.2. Vaginal Environment

For the in vivo studies, G2-S16 PCD was formulated as a water-based gel, as gels appear as optimal formulations to ensure that the microbicide begins to act quickly and that its application is simple. This formulation also shows a high application consistency, thus, ensuring its easy spreading. Moreover, gel viscosity and elasticity enhance drug stability and retention. The vehicle used is a hydroxyethyl-cellulose (HEC) geland and the active pharmaceutical ingredient is G2-S16 PCD. A total of 3% weight/volume (*w*/*v*) of G2-S16 PCD was mixed in 2% (*w*/*v*) of HEC, which is biocomplatible with normal human vagina (Table 1). HEC was used for the different applications, due to its efficiency in absorbing and retaining large quantities of water, while keeping its mechanical and physical conformation. Although G2-S16 PCD microbicide was formulated as a gel, it could be formulated as a cream, film, or vaginal ring.

Previously, G2-S16 PCD was observed to not inhibit beneficial bacterial growth in any selected species in vitro. Due to the physiological relevance of a normal vaginal microbiota, the effect of G2-S16 on this microenvironment was further assessed as a proof-of-concept that would eventually lead to human clinical trials. G2-S16 PCD was tested to check if it would alter the microbiota in BALB/c mice, keeping in mind that 3% SPL7013 showed signs of inflammation and epithelial damage over time, in a phase I clinical trial [115]. BALB/c mice were treated vaginally with 3% G2-S16 PCD gel, and vaginal lavages were obtained before the G2-S16 PCD treatment and at the day of sacrifice. Total nucleic acid extraction for bacteria in mice vaginal lavages was performed. Results were obtained by 16S ribosomal RNA (rRNA) sequencing and the samples were used to obtain the genomic libraries. Subsequent sequencing was performed through the Nextera Illumina technology. G2-S16 PCD did not modify abundance of several species of vaginal microbiota, neither at the genus nor at the phylum level, when compared to the untreated mice [119]. G2-S16 PCD selected concentration was 50-fold higher than one inhibiting R5-HIV_NLAD8_, and no inhibition in beneficial bacterial growth was detected, suggesting no disruptions in vaginal/intestinal ecology.

### 7.3. Microbicide Activity

The mode of action proposed for topical application is based on formation of a lubricant coat on the epithelial surface, once it is inoculated intravaginally. Once there, the G2-S16 PCD establishes a film or a physical barrier to prevent dissemination of either free-cell HIV or infected cells from local mucosa to regional lymph nodes. Testing infection assays in “humanized bone marrow-liver-thymus” (h-BLT) mice validated this mechanism [52].

Animal models are critical for the development and implementation of effective microbicides for evaluation in humans. Although non-human primates (NHP) models are used in pre-clinical studies, it is very important to take into account their high costs, limited availability of female animals, inability to employ HIV for challenge studies and tests against HIV drug-resistant, as well as ethical problems related to their use. It is unclear whether NHP models exactly predict what would occur in humans. The best small-animal models for HIV are humanized mice, specifically, the ones that are genetically immune-compromised and engrafted with human tissues to reconstitute their immune system [120]. Human-BLT mice reproduce the key aspects of human conditions, as their primary cells express normal levels of human receptors and co-receptors, and permit the interaction and subsequent evaluation of HIV infection, using HIV strains [121]. Levels of human CD4 T-cells in HIV-infected h-BLT mice, dramatically decreased during the course of infection, and maintained levels similar to those seen in HIV^+^ h-BLT mice (HEC alone) [122]. In vivo results using h-BLT mice offer strong support to conduct preliminary large-scale screenings in a cost-effective manner [123,124,125]. When h-BLT mice were pre-treated with topical 3% G2-S16 PCD and then vaginally exposed to R5-HIV_JR-CSF_, G2-S16 PCD was found to efficiently prevent vaginal HIV transmission at 84% level (Table 1). These results are encouraging in a model that showed 100% transmission, especially considering that HIV sexual transmission in humans is very inefficient, as it requires time and hundreds of sexual viral exposures, for an organism to become infected [126].

In conclusion, a vaginal/rectal microbicide must display activity against most HIV strains, and other sexually transmitted pathogens that act as a direct virucidal, retain the activity for several hours in the presence of vaginal fluids over a broad pH range, must not fail to prevent semen-exposed HIV infection, should not leak immediately after application, should not accumulate in order to avoid toxicity effects, should not affect normal vaginal microflora, and the structural integrity of vaginal (or rectal) mucosal epithelium must be odorless, colorless, tasteless, stable at higher temperatures, and compatible for use with the male latex condom. Microbicides must have an excellent stability, should be easy to use, show a long shelf-life and be inexpensive and readily accessible. G2-S16 dendrimer could be a new innovative and promising microbicide, meeting all the aforementioned criteria and very close to a clinical trial that could contribute to the development of multi-purpose interventions to prevent HIV infection in women.

**Table 1:** Articles referring G2-S16 activity against HIV.

Date	Assay	Title	Reference
2012	In vitro	Synthesis of polyanionic carbosilane dendrimers and initial evaluations for them to be considered for biomedical applications.	[56]
2012	In vitro	G2-S16 blocks activated PBMC and epithelial cells infection by HIV and/or HIV-2 and partially prevents HIV from crossing through a trans-epithelial monolayer in vitro.	[55]
2012	In vivo	In a 14-day repeat dose experiment using rabbit vagina model, the formulation showed a minimal level of vaginal irritation.	[55]
2013	In vitro	G2-S16 showed a synergistic profile with MRV against CCR5 and dual tropic HIV.	[102]
2015	In vitro	High biosafety demonstrated in human epithelial cell lines derived from uterus and vagina, as well as in PBMC.	[57]
2015	In vitro	Triple combination of G2-S16 with MRV/TDF and other PCD were studied.	[101]
2015	In vitro	G2-S16 PCD showed high anti-HIV-2 activity.	[116]
2015	In vivo	No vaginal irritation was detected in BALB/c mice after two consecutive applications for 2 days with 3% G2-S16 PCD.	[116]
2015	In vivo	G2-S16 PCD prevents vaginal transmission of HIV in humanized BLT mice by 85%.	[52]
2016	In vitro	Mechanism of action of G2-S16 PCD: multifactorial and non-specific function, blocking of gp120/CD4 interaction, acting on the virus and inhibiting the cell-to-cell HIV transmission.	[100]
2016	In vitro	G2-S16 PCD is highly active against HIV, preventing the infection of Treg, and is able to protect Treg from Foxp3 down regulation induced by HIV infection.	[50]
2016	In vitro	G2-S16 PCD blocks HIV infection in presence of SEVI.	[91]
2017	In vitro	High biosafety in Vero cells, HEC1A and VK-2 cell lines.	[67]
2017	In vivo	G2-S16 PCD biodistribution and biosafety after 7 days of daily administration in BALB/c mice.	[67]
2018	In vitro	G2-S16 PCD destabilizes the GP120-CD4 Complex, thus blocking HIV entry and cell to cell fusion.	[103]
2019	In vivo	G2-S16 PCD, in combination with Platycodin D, appears as a vaginal microbicide candidate with contraceptive activity.	[77]
2019	In vitro	Combination of G2-S16 PCD/dapivirine antiretroviral as a new HIV microbicide.	[76]
2019	In vitro	G2-S16 PCD does not generate resistances in MT-2 cell line.	[97]
2019	In vitro	G2-S16 PCD dendrimer microbicide does not interfere with the vaginal immune system.	[75]
2020	In vivo	G2-S16 dendrimer does not modify healthy mouse vaginal microbiome	[119]

## Abbreviations

7AAD	7-Aminoactinomycin D
ARV	Antiretrovirals
CTC	Cell To Cell
DC	Dendritic Cell
DPV	Dapivirine
DC-SIGN	DC-specific intercellular adhesion molecule-3-grabbing non-integrin
FGT	Female Genital Tract
GEC	Genital epithelial cells
HIV	Human Immunodeficiency Virus
HSV-2	Herpes Simplex Virus 2
HSPG	Heparan sulfate proteoglycans
HEC	Hydroxyethyl-cellulose
h-BLT	Humanized bone marrow-liver-thymus
iDC	Immature Dendritic Cell
IL	Interleukin
LDH	Lactate Deshidrogenase
MTT	3-(4,5-dimethyl-2-thiazolyl)-2,5-diphenyl-2H-tetrazoliumbromide
MØ	Macrophage
NNRTT	Non-nucleoside reverse transcriptase inhibitors
NHP	Non-human Primates
PAP	Prostatic acid phosphatase
PAMP	Pathogen-associated Molecular pattern
PCD	Polyanionic Carbosilane Dendrimer
RNA	Ribonucleic Acid
SEVI	Semen-derived Enhanced of Viral Infection
STI	Sexual Transmission Infections
TFV	Tenovofir
TLR	Toll-Like Receptor
UNAIDS	Joint United Nations Program on HIV/AIDS
VOICE	Vaginal and Oral Interventions to Control the Epidemic
